# Omadacycline in the treatment of severe Q fever pneumonia during an influenza epidemic: a case report with literature review

**DOI:** 10.3389/fmed.2025.1626115

**Published:** 2025-08-18

**Authors:** Chuanhua Nie, Yuan Zhu, Huajuan Zhou, Xinmin Zhu, Shaohua Hu, Xiaoli Zhao, Xiaohua Zhong, Fengfei Qian, Miao Yu, Qiuting Jiang

**Affiliations:** Department of Respiratory and Critical Care Medicine, Hangzhou Linping Hospital of Integrated Traditional Chinese and Western Medicine, Hangzhou, Zhejiang, China

**Keywords:** Q fever, influenza, targeted high-throughput sequencing (tNGS), omadacycline, case

## Abstract

Q fever, caused by *Coxiella burnetii* (Q fever *rickettsiae*), is a zoonotic disease with a natural reservoir and has been reported in many countries and regions. Its clinical presentation is non-specific and easily confused with other infectious or non-infectious diseases. Conventional diagnostic methods, such as respiratory specimen culture, often fail to yield conclusive results, increasing the risk of misdiagnosis. This case involves a 78-year-old male patient from Zhejiang Province, China, who presented with fever as the primary complaint and developed severe pneumonia. The diagnosis of H1N1 influenza co-infection with Coxiella burnetii was confirmed by bronchoalveolar lavage fluid targeted high-throughput sequencing (tNGS). Following antiviral treatment with Maraviroc and antibiotic therapy with omadacycline tosilate (a novel tetracycline-class drug) for Coxiella burnetii infection, the patient’s clinical symptoms improved, biochemical markers normalized, and pulmonary imaging showed resolution. This case highlights the potential of tNGS to improve the detection rate of mixed infections in cases of severe pneumonia of unknown etiology. The novel tetracycline drug, such as omadacycline, has demonstrated efficacy against Q fever rickettsial pneumonia, offering a new perspective for clinical diagnosis and treatment.

## Introduction

Q fever, also known as Query Fever, is a rare zoonotic disease caused by Coxiella burnetii (Q fever rickettsia). It was first identified and diagnosed in slaughterhouse workers in Australia in 1937, with outbreaks reported in regions such as the Netherlands, Egypt, Algeria, and others (1–3). Coxiella burnetii is a gram-negative intracellular pathogen, and its clinical manifestations can vary significantly following infection. Approximately 60% of infected individuals experience asymptomatic latent infections (1). However, during the Q fever outbreak in the Netherlands from 2007 to 2009, 86% of hospitalized patients with acute Q fever exhibited signs of pneumonia (4).

The main clinical symptoms and signs of acute Q fever include high fever, cough, dyspnea, and abnormal lung auscultation, often accompanied by extra-pulmonary signs such as myalgia, arthralgia, sore throat, nausea, vomiting, abdominal pain, diarrhea or constipation, and relative bradycardia (5). Approximately 4%–5% of Q fever cases may progress to chronic persistent infection, The prognosis is generally poor (6), Due to the lack of specificity in its clinical presentation, Q fever is often misdiagnosed or missed. The primary route of transmission to humans is through the inhalation of aerosolized pseudospores (7). Human-to-human transmission is extremely rare.

Recently, our hospital admitted a 78-year-old male patient who presented with high fever, cough, and fatigue. Laboratory tests revealed co-infection with H1N1 influenza A. It is worth noting that early acute Q fever often presents with flu-like symptoms (8), which can easily lead to diagnostic confusion. In this report, the author will review and analyze this case of mixed infection to highlight the key points in the clinical diagnosis and treatment of Q fever.

## Medical record

**FIGURE 1:**
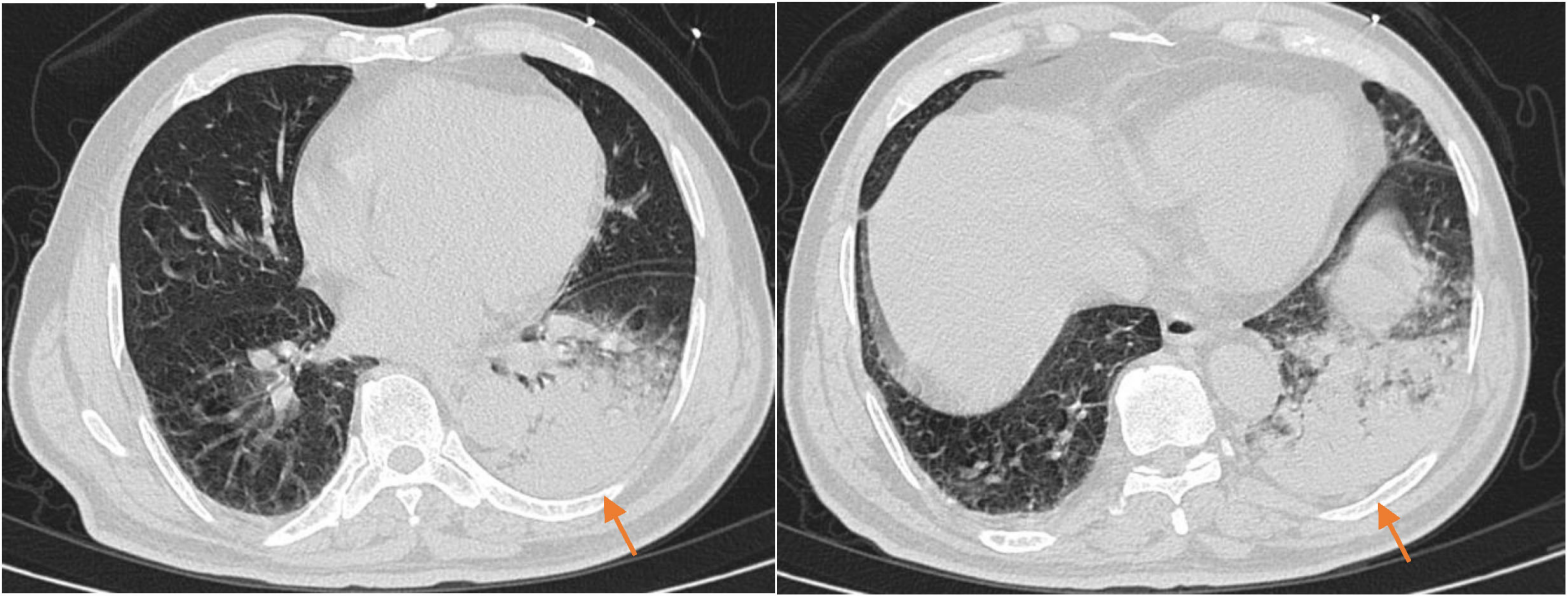
The chest CT scan before admission (2025-02-12) showed a patchy high-density shadow in the left lower lung, with prominent local margin infiltration, partial consolidation, and visible bronchial air sign.

Present Illness History: A 78-year-old male farmer from a rural area in Zhejiang Province was admitted to the Respiratory Department at our hospital on February 12, 2025, with a chief complaint of “fever, accompanied by cough and fatigue for 4 days.” Four days before admission, the patient developed chills and fever without an apparent cause. His temperature peaked at 39.0°C, and he experienced paroxysmal coughing with minimal white sputum production, significant fatigue, reduced appetite, and chest tightness with shortness of breath during exertion. Symptoms did not improve significantly with rest at home, prompting him to visit the emergency department of our hospital. Upon arrival, his oxygen saturation, measured by pulse oximetry, was 89%. The following tests were conducted:

Chest CT: Left lower lung infiltrative changes (Figure 1).

Laboratory test results were as follows: Pneumonia Mycoplasma nucleic acid & Type A Influenza Virus antigen: Negative, Table 1 shows the results of other laboratory tests.

**TABLE 1:** Laboratory test results at different time points for this patient.

Laboratory test	Normal range	First day of hospitalization	Second day of hospitalization	Fourth day of hospitalization	After 1 week of hospitalization	One day before discharge
**Routine bloodwork**						
WBC (×10^9^/L)	3.5–9.5	7.0	7.3	6.3	7.2	7.0
Neutrophil (%)	40–70	84.9	85.6	82.4	77.0	65
PaO_2_	80–100	58.6	78.1	92.1	98	/
PaO_2_/FiO_2_	350–500	182.7	156.2	262.9	357.2	/
**Inflammatory index**						
C-reactive protein (mg/L)	0–8	162.2	182.6	56.0	9.0	3.2
Procalcitonin (ng/mL)	<0.5	/	1.06	0.55	/	/
IL-6 (pg/mL)	0–7	/	98.5	8	2	/
**Biochemical indexes**						
ALT (U/L)	0–49	27	76	41	33	27
AST (U/L)	0–50	44	92	36	26	23
LDH (U/L)	0–248	204	280	165	142	156
D-dimer (mg/L)	0–550	1065	1757	957	682	476
Scr (mmol/L)	64–104	98	102	83	90	94
CK (U/L)	0–164	235	210	150	123	156
CK-MB (ng/mL)	0–5	7.91	3.91	3.14	2.52	3.12
K (mmol/L)	3.5–5.3	3.57	3.4	3.2	4.0	4.4

WBC, white blood cell; IL-6, interleukin 6; ALT, alanine aminotransferase; AST, aspartate aminotransferase; LDH, lactate dehydrogenase; Scr, serum creatinine; CK, creatine kinase; CK-MB, creatine kinase muscle and brain isoenzyme.

Past Medical History: The patient has a history of “primary hypertension” and “benign prostatic hyperplasia”, with long-term medication use to manage these conditions, which are currently under control.

The patient primarily follows a vegetarian diet and denies any contact with epidemic or high-risk areas, but a detailed personal history revealed that the patient’s neighbor raises goats. There is no history of smoking, alcohol use, or radiation exposure, nor any history of mental illness or unprotected sexual activity. The patient has no significant marital, reproductive, or family history.

Preliminary Diagnosis: Severe pneumonia.

Physical examination upon admission: Temperature: 37.9°C, Pulse: 96 beats/min, Respiratory rate: 28 breaths/min, Blood pressure: 171/88 mmHg, Blood glucose: 10.0 mmol/L, Oxygen saturation: 94% (with nasal cannula oxygen supplementation, oxygen concentration 35%). The patient was transported to the ward on a stretcher, fully conscious, with slightly altered mental status, rapid breathing. No cyanosis in the mouth or lips, yellow discoloration of the skin or sclera, rash, subcutaneous bleeding, or palpable superficial lymph nodes were observed Coarse breath sounds were heard in both lungs, moist rales auscultated in the left lower lung. The heart rhythm was regular, with no pathological murmurs detected. Abdominal and neurological examinations were unremarkable.

Auxiliary examinations upon admission: Arterial blood gas analysis recheck the following morning (under high-flow nasal cannula oxygen therapy with a flow rate of 40 L/min and oxygen concentration of 50%): B-type natriuretic peptide, thyroid function, routine urinalysis, routine stool test & occult blood showed no significant abnormalities, other laboratory tests are presented in Table 1, Liver, gallbladder, pancreas, spleen, and bilateral kidneys, ureters, bladder, prostate ultrasound: fatty liver, liver cyst, splenomegaly, benign prostatic hyperplasia with calcification; Bilateral lower extremity arterial and venous ultrasound: bilateral lower extremity arterial sclerosis with multiple plaque formations, unobstructed blood flow in the deep venous system of both legs. Cardiac 2D echocardiography: aortic sclerosis, mild mitral and tricuspid regurgitation, LVEF 69%. ECG: sinus rhythm, normal range ECG.

**FIGURE 2:**
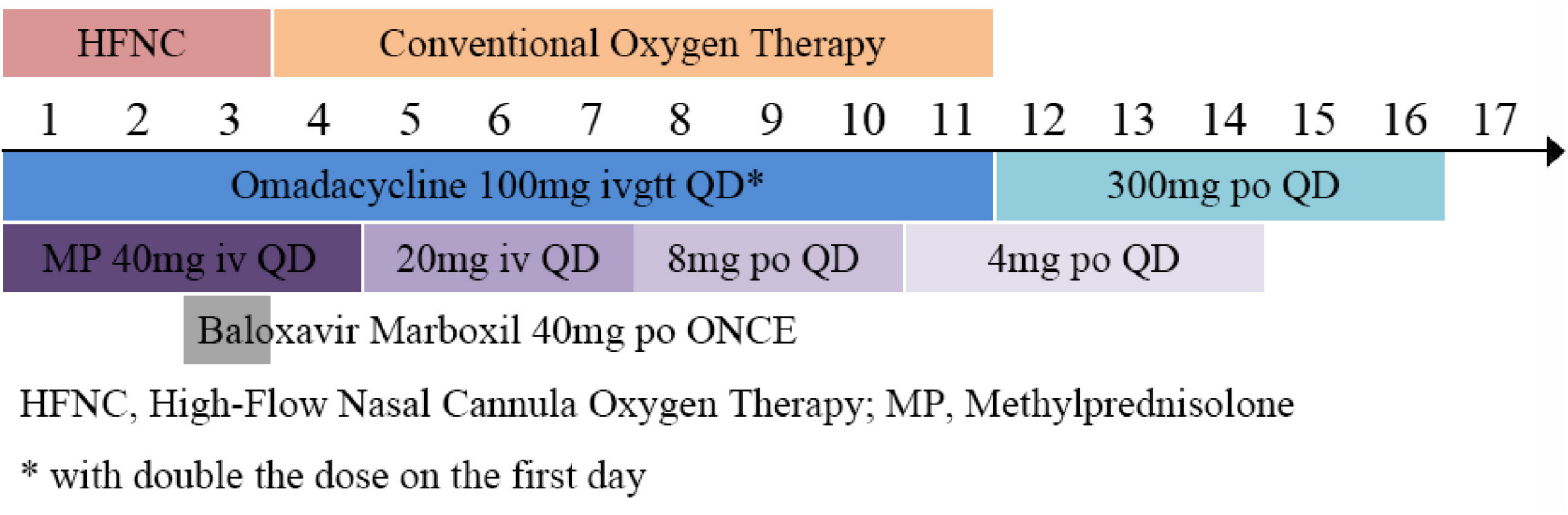
Therapeutic management flow chart.

Treatment Plan: The patient was admitted with the chief complaint of “fever, cough, and fatigue for 4 days. The oxygenation index (OI) was 156 (<250 mmHg), the respiratory rate was 28 breaths/min, and chest imaging showed multiple lung lobe infiltrations (Figure 1), meeting the diagnostic criteria for severe pneumonia. Blood and sputum cultures were taken, and the following treatments were initiated (Figure 2):

High-flow nasal humidified oxygen therapy (oxygen concentration 50%, flow rate 40 L/min),
Omadacycline tosylate injection (0.1 g once daily, intravenous infusion, with double the dose on the first day) for anti-infection,
Thymosin peptide (1.6 mg, subcutaneous injection twice a week) for immune modulation,
Methylprednisolone injection (40 mg once daily by intravenous push, with gradual dose reduction after 4 days) for anti-inflammation,
Ambroxol hydrochloride injection (30 mg once daily by intravenous infusion),
Symptomatic treatment, including fluid supplementation.

On the second day of hospitalization, an electronic bronchoscopy was performed and showed clear bronchi with a small amount of yellow secretion aspirated from the left lower lobe, which was lavaged and brushed for testing. The bronchoalveolar lavage fluid (BALF) was sent for tNGS testing, which identified *Coxiella burnetii* (sequence count 26), H1N1 influenza virus (sequence count 58194), and *Staphylococcus aureus* (sequence count 22). Based on the pathogen detection results, the patient continued receiving omadacycline for anti-infection treatment and was given Baloxavir Marboxil (40 mg, oral, once) for anti-influenza treatment.

From the second day of hospitalization, the patient’s peak temperature decreased, and symptoms such as cough and fatigue showed improvement. On the fourth day of hospitalization, oxygen therapy was switched to a low-flow nasal cannula (2 L/min). After 7 days, we have re-examined the laboratory tests (Table 1).

**FIGURE 3:**
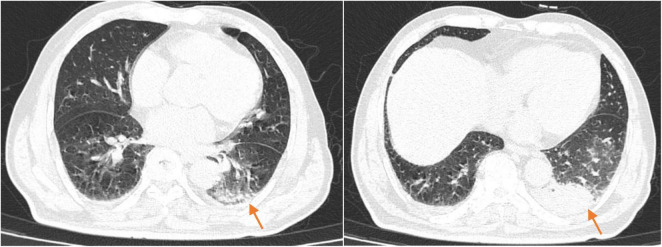
The chest CT on reexamination (2025-02-23) showed a high-density shadow in the left lower lobe, with a significantly reduced area compared to the previous scan.

**FIGURE 4:**
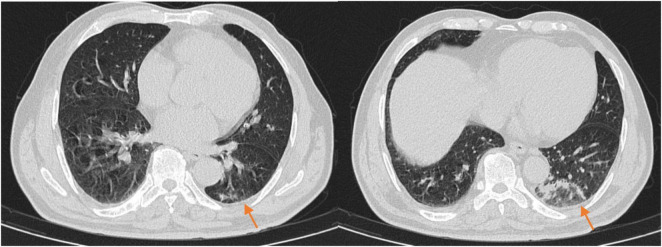
The chest CT 1 month after discharge (2025-03-23) showed patchy and linear high-density shadows in both lungs, with a significant reduction in size compared to the previous scan.

On the 10th day of treatment, a recheck of chest CT showed significant absorption of the infection focus compared to previous scans (Figure 3), and all laboratory parameters have normalized (Table 1). The patient was discharged on day 11 after significant improvement. After discharge, the patient continued oral omadacycline (300 mg once daily) for 5 days before discontinuing the medication (Figure 2): a follow-up was conducted 1 month after discharge, and the patient’s symptoms had completely resolved. A repeat chest CT showed significant absorption of the exudates (Figure 4).

## Literature review

A search for the term “Q Fever” was conducted in the PubMed database, covering the period from January 2000 to April 2025. A total of 3,646 English-language articles were retrieved. Relevant studies were selected and reviewed to analyze the microbiological characteristics of *Coxiella burnetii*, epidemiological features, clinical manifestations in patients, laboratory tests, imaging findings, treatment, and prognosis.

(1) Microbiological characteristics

Q fever is a zoonotic disease caused by *Coxiella burnetii*, which is widely distributed around the world. *Coxiella burnetii* is a gram-negative intracellular bacterium (1) known for its high environmental resistance, allowing it to survive for months to years under conditions of high temperatures, dryness, and UV radiation. The bacterium has two main forms in its life cycle: the small cell variant (SCV) and the large cell variant (LCV), also referred to as phase I and phase II forms, respectively.

During the initial stages of infection, *Coxiella burnetii* enters immune cells such as monocytes and macrophages, where it replicates in the form of SCV. This form primarily expresses phase I antigens and is highly virulent. Under low-nutrient or adverse conditions, *Coxiella burnetii* transforms into the LCV form (phase II), which has reduced virulence, loses some surface antigens, and expresses phase II antigens. The LCV form mainly participates in replication and transmission (9). The intracellular parasitic nature of *Coxiella burnetii* enables it to evade the host’s immune system and form a specialized parasitic vacuole within host cells for replication. This adaptability makes it a pathogen that is difficult to eliminate (10).

(2) Epidemiological characteristics

Q fever is a globally distributed disease, the primary hosts of *Coxiella burnetii* are cattle, sheep, and goats. However, recent reports indicate that an increasing number of animals, including domestic mammals, marine mammals, reptiles, ticks, and birds, have been identified as transmitters of this pathogen (11). The pathogen can be present in the urine, feces, and milk of infected animals. In epidemiological studies, outbreaks of Q fever are often associated with animal parturition or slaughter activities (12). Additionally, Q fever typically occurs in the spring and summer, when animal parturition and daily activities are more frequent, significantly increasing the risk of human infection (13).

Besides inhalation of aerosolized pseudospores, other reported cases include hospital-acquired infections caused by vaginal secretions or placental tissue (14), infections following bone marrow transplantation (15), infections via breastfeeding (16), household cluster infections (17), and hospital-acquired infections during childbirth or autopsy (18).

(3) Clinical manifestations

The clinical manifestations of Q fever vary depending on the severity of the infection and the immune status of the patient. Among symptomatic patients, Q fever is traditionally classified into acute and chronic forms. Acute Q fever typically presents with non-specific symptoms, including fever, headache, fatigue, myalgia, and nausea. Some patients may develop signs of pneumonia or hepatitis (19). Infection during pregnancy may lead to adverse pregnancy outcomes, such as miscarriage or preterm birth (20). Chronic persistent infection, with symptoms that may include persistent fever, weight loss, night sweats, signs of heart or large vessel vascular lesions, and prosthetic infections (6), and the most life-threatening complication is endocarditis (21).

In recent years, as case data has accumulated, researchers have found that relying solely on serological criteria to diagnose “chronic Q fever” is insufficiently accurate (22). The serum phase I IgG titer can vary significantly due to differences in *Coxiella burnetii* strains and patient clinical characteristics. This term tends to conflate different clinical entities that require distinct prevention, diagnosis, and treatment strategies. The natural course of Q fever infection is similar to that of tuberculosis; the primary infection may be symptomatic or asymptomatic, and, if left untreated, it may progress to a persistent infection, leading to various localized manifestations depending on the susceptibility of the host. Based on the polymorphic manifestations of *Coxiella burnetii* infection, researchers have proposed separate diagnostic criteria for persistent infection foci at different locations, gradually moving away from the misleading and controversial definition of “chronic Q fever”.

(4) Laboratory tests

A complete blood count typically shows a normal white blood cell count with elevated C-reactive protein levels, and elevated liver enzymes are also common (4). The laboratory diagnosis of Q fever includes serological tests, bacteriological cultures, and molecular biological methods. However, bacteriological cultures have high biosafety requirements, making serological testing the primary diagnostic method in clinical practice (11). An IgG titer > 1:2000 or an IgM titer > 1:50 suggests acute Q fever (23). Additionally, molecular biological tests, such as polymerase chain reaction (PCR), can detect *Coxiella burnetii* DNA in various clinical samples, including blood, sputum, bronchoalveolar lavage fluid, heart valve, or other surgical tissue biopsy specimens. The advantage of PCR is its ability to detect *Coxiella burnetii* before seroconversion in primary infected patients. The disadvantage is that the bacterial concentration in blood is low, leading to a lower PCR positivity rate, especially after antibiotic treatment (22). Metagenomics next-generation sequencing (mNGS) can help diagnose *Coxiella burnetii*, particularly in patients with atypical clinical presentations or unclear epidemiological exposure evidence (24). However, it has drawbacks, such as being time-consuming and expensive.

(5) Imaging studies

Pneumonia is a common manifestation of acute Q fever, but chest X-rays are often insufficient to distinguish Q fever pneumonia from other diseases. The most common imaging feature is localized involvement of a lung segment or lobe, although this is not specific (25). On CT scans, Q fever pneumonia typically appears as segmental, patchy, or lobar consolidation. More than half of the patients exhibit involvement of more than one lung lobe, and in immunocompromised patients, abscesses and other necrotic changes may also be observed (26). 18F-FDG Positron Emission Tomography/Computed Tomography (18F-FDG PET/CT) is commonly used to help localize the sites of infection in patients with persistent infection (22).

(6) Treatment and prognosis

The treatment of Q fever is primarily based on antibiotics. For rapidly progressing Q fever pneumonia, early treatment is essential, and tetracycline drugs are recommended as first-line agents (11). The first-line treatment for acute Q fever is doxycycline, with an adult dose of 100 mg every 12 h (either orally or intravenously). The recommended dose for children is 2.2 mg/kg every 12 h (with a maximum dose of 200 mg per day), with a treatment duration of 2–3 weeks. For pregnant women, co-trimoxazole is recommended, with a dose of sulfamethoxazole 800 mg/trimethoprim 160 mg every 12 h. Chronic infection of the placenta and endometrium may occur during pregnancy, so treatment should be continued at least until delivery (11). For patients who are allergic or intolerant to doxycycline, moxifloxacin, clarithromycin, trimethoprim-sulfamethoxazole, and rifampin are alternative treatment options (27, 28).

The prognosis for Q fever is generally favorable in most acute cases. However, chronic Q fever can lead to severe complications, such as endocarditis, and is associated with a poor prognosis. To date, the combination of doxycycline and hydroxychloroquine remains the preferred treatment regimen for chronic Q fever (29). Patients with chronic endocarditis should undergo serological monitoring for at least 5 years due to the risk of relapse (30). Therefore, timely diagnosis and treatment, especially the use of antibiotics within 3 days of symptom onset, are crucial to improve the prognosis (31).

## Discussion

Based on an epidemiological review of this case, the patient is an elderly male living alone in a rural area, with a predominantly vegetarian diet and no history of consuming dairy products such as cow’s milk or goat’s milk. He has no recent travel history. A detailed personal history revealed that the patient’s neighbor raises goats, and there was a recent history of goat abortion. However, no rigorous disinfection measures were implemented afterward. The patient also has a habit of walking outdoors. Therefore, there is a strong suspicion that the infection was caused by inhalation of contaminated aerosol particles. This mode of transmission should be highlighted in rural public health control efforts.

In laboratory diagnosis, *Coxiella burnetii* (the causative agent of Q fever) cannot be cultured using conventional laboratory culture techniques, making microbial culture challenging. Serology remains the most commonly used method for detecting Q fever (23), however, it lacks specificity and its interpretation has significant regional variations, which may affect Q fever diagnosis (22). In recent years, there have been case reports highlighting the use of mNGS (metagenomic next-generation sequencing) technology for Q fever diagnosis (32–34), though its clinical application is somewhat limited due to issues with cost and time consumption. Bronchoalveolar lavage fluid tNGS detection is a novel diagnostic method that captures and analyzes pathogen DNA and RNA sequences in samples, performing high-throughput sequencing of target regions. It is efficient, cost-effective, and significantly reduces the potential for human contamination due to its high automation (35). This method has been widely applied in the etiological diagnosis of severe pneumonia (36). In the diagnosis and treatment of severe pneumonia, early identification and the use of anti-infective drugs targeting pathogens can significantly improve patient survival rates (37), tNGS technology plays a crucial role in the early, precise treatment of pulmonary infections. Currently, existing literature has reported that tNGS can serve as a diagnostic criterion for rickettsial diseases (38). In this case, after the pathogen was identified with the assistance of tNGS testing of BALF, the specimen was sent to the Zhejiang Provincial Center for Disease Control and Prevention in Hangzhou for PCR validation, ensuring the accuracy of the diagnosis (39). Furthermore, it also provided supporting evidence for the potential use of tNGS in diagnosing Q fever. In summary, given symptoms of the patient, laboratory data, imaging, tNGS detection of *Coxiella burnetii* in bronchoalveolar lavage fluid and the validation of PCR, the case was definitively diagnosed.

In terms of treatment, tetracyclines are internationally recommended as first-line drugs (11). Tetracyclines exert their antibacterial effect primarily by binding to the A site of the bacterial ribosomal 30S subunit, thereby inhibiting bacterial protein synthesis. Omadacycline tosylate is a novel aminomethylcycline antibiotic and a derivative of minocycline. It features an aminocarbamate group introduced at the C7 position and a glycyl modification at the C9 position. These structural modifications help reduce bacterial resistance, expand the antimicrobial spectrum, and improve drug permeability, tissue distribution, and metabolic stability. The tosylate group, which serves as the salt base of the drug, enhances stability and solubility without affecting its antimicrobial activity, making it suitable for industrial-scale production. The antimicrobial spectrum of omadacycline covers gram-positive, gram-negative, atypical pathogens, and anaerobes, demonstrating great efficacy and safety for the treatment of community-acquired bacterial pneumonia (40). It does not require dosage adjustments in patients with liver dysfunction (41). The drug was approved by the US FDA in 2018 for the treatment of acute bacterial skin and skin structure infections and community-acquired pneumonia in adults (42), and was launched in China in 2021, although clinical experience remains limited. The patient in this case met the diagnostic criteria for severe pneumonia upon admission (37) and was complicated by liver dysfunction. Omadacycline was selected for empirical anti-infection therapy. Subsequent bronchoalveolar lavage fluid tNGS indicated a co-infection with *Coxiella burnetii* and influenza A virus, but the antibiotic regimen was not altered. To date, there have been no reported clinical cases of using novel semi-synthetic tetracycline antibiotics to treat Q fever, making this study the first of its kind, with a favorable treatment outcome, providing a valuable clinical example.

In terms of the treatment regimen, this case initially involved a 10-day course of intravenous infusion with the injectable form (100 mg/day, doubled on the first day). After discharge, the treatment was switched to an oral formulation for 5 days of sequential anti-infective therapy (at a dose of 300 mg/day). The difference in dosage between the injectable and oral formulations is due to their different bio availabilities (100 and 34.5%, respectively). The Cmax of a 300 mg oral dose is comparable to that of a 100 mg intravenous dose, although the AUC_0_-∞ is higher with intravenous administration. Intravenous administration is preferred in the early stages of treatment, while sequential therapy can be switched to the oral form to reduce the risk of adverse effects, such as phlebitis (43).

The clinical manifestations, laboratory tests, and imaging findings of acute Q fever pneumonia lack specificity and are difficult to distinguish from common community-acquired pneumonia. In this case, the patient’s illness occurred during the influenza A epidemic in Zhejiang Province. Symptoms such as fever and fatigue, which resemble flu-like symptoms, could potentially mislead the diagnosis. Auxiliary examinations revealed elevated serum C-reactive protein levels and lobar pneumonia changes on chest CT. Upon admission, the patient developed respiratory failure and liver dysfunction. The overall condition progressed rapidly, and the patient’s epidemiological history was unclear at the time of initial diagnosis. Q fever cases are rare in Zhejiang, making the diagnosis easy to miss. After the diagnosis was rapidly clarified using bronchoalveolar lavage fluid tNGS, the patient was treated with omadacycline, which yielded a good therapeutic outcome. This case demonstrates that for patients with pulmonary infections who fail initial empiric treatment, early bronchoalveolar lavage fluid tNGS testing to identify the pathogen and guide antimicrobial selection can significantly improve prognosis, especially when considering infections caused by atypical pathogens.

In summary, the clinical manifestations and auxiliary examination results of Q fever lack specificity, with pneumonia being a common clinical presentation of acute Q fever. In terms of diagnosis, targeted next-generation sequencing (tNGS) of bronchoalveolar lavage fluid has significant value in diagnosing *Coxiella burnetii* infection. Regarding treatment, the novel tetracycline antibiotic omadacycline has been shown to effectively improve the prognosis of patients with severe Q fever pneumonia, offering an innovative and safe treatment option for this patient population.

## Data Availability

The original contributions presented in the study are included in the article/Supplementary materials. Further inquiries can be directed to the corresponding author.
