# Supplementary material for: Omadacycline in the treatment of severe Q fever pneumonia during an influenza epidemic: a case report with literature review
Source: Front Med (Lausanne). 2025 Aug 18;12:1626115. doi: 10.3389/fmed.2025.1626115 (PMC12401012; doi:10.3389/fmed.2025.1626115)
Supplement: Supplementary file 1 [file Data_Sheet_1.pdf]

Clinical Lab Tests Report

Client information

**Client**	NAME: ***	Gender: Male	Age: 78year(s)
	In-Patient NO.: 1005103058		Bed NO.: 1033
**Specimen**	Barcode: 0558000947	Test NO.: T16165	Specimen Type: Bronchoalveolar lavage fluid
	Collection Time: 2025-02-14	Receiving Time: 2025-02-14 11:00:52	Hospital ID:
**Sent by**	Hospital: 杭州市临平区中西医结合医院		
	Division: 呼吸科		Physician:
**Clinical**	Clinical Diagnosis:		
**Test**	Test Name: Targeted NGS of multiple pathogens in respiratory system		Test Method: multiplex targeted PCR-NGS

Pathogenic Microorganism Detected

Generic	Species	Rpk	Copies/mL Estimated	Pathogenicity classification
**1.List of special pathogens (Mycobacterium , Myco plasma , Chlamydia , etc .)**				
Coxiella	*Coxiella burnetii*	26	<1.0x10^3	A
**2.List of Bacteria**				
Haemophilus	*Haemophilus influenzae*	31	<1.0x10^3	A
Staphylococcus	*Staphylococcus aureus*	22	<1.0x10^3	A
Acinetobacter	*Acinetobacter baumannii*	269	<1.0x10^3	B
Klebsiella	*Klebsiella variicola*	148	1.4x10^3	B
Fusobacterium	*Fusobacterium nucleatum*	69	<1.0x10^3	C
Streptococcus	*Streptococcus anginosus group*	52	<1.0x10^3	C
**3.List of Fungi**				
Negative				
**4.List of Virus**				
Alphainfluenzavirus	*Influenza A virus(H1N1pdm09)*	58194	>1.0x10^6	A
Lymphocryptovirus	*Epstein-Barr virus*	30	<1.0x10^3	C

Rpk: Every 100K of the original sequence contains the number of the microorganism, and the higher the number of the Rpk, the higher the certainty that the sample contains the microorganism.

Copies/mL: The content of microorganisms in samples was calculated by bioinformatics methods. The results are not absolute quantitative and are only used for clinical reference.

Pathogenicity classification A: In this specimen type, it is an obligate pathogenic microorganism or a common pathogenic microorganism in clinical practice.

Pathogenicity classification B: In this specimen type, it is opportunistic pathogenic microorganism, which may cause infection under the condition of systemic or local immunodeficiency/impairment/deficiency. Please comprehensively consider whether it is pathogenic according to the patient's clinical situation.

Pathogenicity classification C: In this specimen type, it is a common colonizing microorganism of human body. In special circumstances, it may migrate to non-colonizing sites and cause infection.

NOTE: The above classification of microorganism is for clinical reference only, and the final definition of microorganism is subject to clinical practice.

Drug Resistance Gene Results

Drug resistance gene mutation sites	Rpk

Quality Control

Total sequence	*Q30	Internal control	Positive Control	Negative Control	Blank Control
Acceptable	Acceptable	Acceptable	Acceptable	Acceptable	Acceptable

NOTE：The above quality control information indicates that the sequencing data of this test is qualified and the results are credible.

*Q30：The Q30 ratio is the percentage of bases that are more than 99.9% correct. The Q30 ratio is the percentage of bases that are more than 99.9% correct。

Description

Signature

HANGZHOU KINGMED FOR CLINICAL LABORATORY

#NAME?

Clinical Lab Tests Report

Name:史发松
Barcode:0558000947
Test NO.:T16165

Supplementary table 1. The panel of our tNGS detection for common respiratory pathogens.

Primary category (n)	Secondary category	Species Name
Virus (81)	DNA virus	*BK polyomavirus, JC polyomavirus, WU Polyomavirus, Human bocavirus, Human bocavirus 1,Human bocavirus 2, Human bocavirus 3, Human bocavirus 4, Cytomegalo Virus, Human parvovirus B19, Epstein-Barr virus, Human mastadenovirus, Human adenovirus 11, Human adenovirus 14, Human adenovirus 1, Human adenovirus 21, Human adenovirus 2, Human adenovirus 34, Human adenovirus 35, Human adenovirus B3, Human adenovirus E4, Human adenovirus 55, Human adenovirus 57, Human adenovirus 5, Human adenovirus 6, Human adenovirus 7, Human mastadenovirus B, Human mastadenovirus C, Human mastadenovirus D, Human herpes virus-6A, Human herpes virus-6B, Human herpes virus-6, Human herpes virus-7, Herpes simplex virus 1, Herpes simplex virus 2, Varicella Zoster Virus*
	RNA virus	*Human coronavirus 229E, Human coronavirus NL63, Influenza A virus, Influenza A virus H1N1, Influenza A virus(H1N1pdm09), Influenza A virus H3N2, Influenza A virus H5N1, Influenza A virus H7N9, SARS-CoV-2, Human coronavirus HKU1, Human coronavirus OC43, Influenza B virus, Echovirus E18, Echovirus E30, Rhinovirus, Rhinovirus A, Rhinovirus B, Rhinovirus C, Enterovirus, Enterovirus A71, Enterovirus A, Enterovirus B, Enterovirus C, Enterovirus D68, Enterovirus D, Coxsackievirus A10, Coxsackievirus A16, Coxsackievirus A2, Coxsackievirus A5, Coxsackievirus A6, Coxsackievirus B3, Influenza C virus, Human metapneumovirus, Measles morbillivirus, Human respiratory syncytial virus A, Human respiratory syncytial virus B, Human orthorubulavirus 2, Human orthorubulavirus 4, Mumps orthorubulavirus, Human respirovirus 1, Human respirovirus 3, Rubella virus, Human parainfluenza virus*
Bacteria (92)	Gram-positive cocci	*Parvimonas micra, Rhodococcus hoagii, Staphylococcus aureus, Staphylococcus lugdunensis, Streptococcus pneumoniae, Streptococcus pyogenes, Streptococcus agalactiae, Streptococcus anginosus group, Streptococcus intermedius, Streptococcus mitis group*
	Gram-positive bacilli	*Mycobacterium xenopi, Mycobacterium gordonae, Mycobacterium tuberculosis complex, Mycobacterium kansasii, Mycobacterium scrofulaceum, Mycobacterium malmoense, Mycobacterium avium, Mycobacterium avium complex, Mycobacterium shimoidei, Mycobacterium szulgai, Mycobacterium asiaticum, Mycobacterium celatum, Mycobacterium simiae, Mycobacteroides chelonae, Mycobacteroides abscessus complex, Mycobacteroides abscessus, Mycolicibacterium smegmatis, Mycolicibacterium fortuitum, Nocardia concava, Nocardia brasiliensis, Nocardia farcinica, Nocardia africana, Nocardia abscessus, Nocardia, Nocardia cyriacigeorgica, Nocardia terpenica, Nocardia otitidiscaviarum, Nocardia nova, Nocardia asteroides, Tropheryma whipplei, Trueperella pyogenes, Corynebacterium striatum, Listeria monocytogenes, Mycobacteroides abscessus subsp. bolletii ,Mycobacteroides abscessus subsp. massiliense, Mycobacteroides abscessus subsp. abscessus, Nocardia transvalensis*
	Gram-negative cocci	*Neisseria meningitidis*
	Gram-negative bacilli	*Acinetobacter baumannii, Acinetobacter junii, Acinetobacter ursingii, Bacteroides fragilis, Bordetella pertussi s, Bordetella parapertussis, Bordetella holmesii, Brucella, Burkholderia mallei, Burkholderia multivorans, Burkholderia pseudomallei, Burkholderia contaminans, Burkholderia cenocepacia, Burkholderia cepacia, Burkholderia cepacia complex, Elizabethkingia anophelis, Elizabethkingia meningoseptica, Enterobacter cloacae complex, Escherichia coli, Fusobacterium necrophorum, Fusobacterium nucleatum, Haemophilus influenzae, Klebsiella variicola, Klebsiella aerogenes, Klebsiella oxytoca, Klebsiella pneumoniae, Legionella bozemanae, Legionella, Legionella micdadei, Legionella pneumophila, Legionella longbeachae, Moraxella catarrhalis, Pasteurella multocida, Proteus mirabilis, Pseudomonas aeruginosa, Serratia marcescens, Stenotrophomonas maltophilia, Acinetobacter calcoaceticus/baumannii complex, Citrobacter freundii comple x, Elizabethkingia*
	Rickettsia	*Coxiella burnetii*
Mycoplasma / Chlamydia, etc(8)	Mycoplasma	*Mycoplasma pneumoniae, Mycoplasma hominis, Ureaplasma parvum, Ureaplasma urealyticum*
	Chlamydia	*Chlamydia pneumoniae, Chlamydia trachomatis, Chlamydia psittaci*
	Leptospira	*Leptospira*
Fungus (43)		*Aspergillus, Aspergillus niger complex, Aspergillus flavus complex, Aspergillus terreus complex, Aspergillus fumigatus, Candida albicans, Candida parapsilosis, Candida orthopsilosis, Candida tropicalis, Cryptococcus gattii, Cryptococcus neoformans, Fusarium, Histoplasma capsulatum, Lichtheimia ramosa, Lichtheimia, Lichtheimia corymbifera, Meyerozyma guilliermondii, Mucor irregularis, Mucor racemosus, [Candida] glabrata, Pichia kudriavzevii, Pneumocystis jirovecii, Rhizomucor, Rhizomucor pusillus, Rhizopus delemar, Rhizopus, Rhizopus oryzae, Rhizopus microsporus, Scedosporium boydii, Scedosporium apiospermum, Scedosporium, Talaromyces marneffei, Trichosporon asahii,Candida auris, Cryptococcus, Mucor, Lomentospora prolificans, Candida, Coccidioides, Cunninghamella, Microascus, Cryptococcus laurentii, Syncephalastrum*
Parasite (1)		*Paragonimus westermani*

Technologist	[illegible]	Reviewer	[illegible]	
Lab:HANGZHOU KINGMED		Report Time:2025-06-25 16:57:36		Collecting Site :杭州市临平区中西医结合医院-呼吸内科
Web:www.kingmed.com.cn		Tel:4001-111-120		
Add:Building 5, Yin Hai Science and Technology Innovational Centre, Xia Sha Community , Qian Tang District, Hangzhou				HZ003KFQZ7J8ACM
